# Development and Implementation of a Multidisciplinary Electronic Discharge Readiness Tool: User-Centered Design Approach

**DOI:** 10.2196/24038

**Published:** 2021-04-23

**Authors:** Angela Keniston, Lauren McBeth, Jonathan Pell Sr, Kasey Bowden, Stephen Ball, Kristin Stoebner, Elaina Scherzberg, Susan L Moore, Jamie Nordhagen, Amanda Anthony, Marisha Burden

**Affiliations:** 1 Division of Hospital Medicine Anschutz Medical Campus University of Colorado Aurora, CO United States; 2 UCHealth Denver, CO United States; 3 Colorado School of Public Health University of Colorado Aurora, CO United States

**Keywords:** user-centered design, stakeholder engagement, health information technology, implementation science, interdisciplinary, teamwork, discharge planning, discharge readiness tool

## Abstract

**Background:**

Typical solutions for improving discharge planning often rely on one-way communication mechanisms, static data entry into the electronic health record (EHR), or in-person meetings. Lack of timely and effective communication can adversely affect patients and their care teams.

**Objective:**

Applying robust user-centered design strategies, we aimed to design an innovative EHR-based discharge readiness communication tool (the Discharge Today tool) to enable care teams to communicate any barriers to discharge, the status of patient discharge readiness, and patient discharge needs in real time across hospital settings.

**Methods:**

We employed multiple user-centered design strategies, including exploration of the current state for documenting discharge readiness and directing discharge planning, iterative low-fidelity prototypes, multidisciplinary stakeholder meetings, a brainwriting premortem exercise, and preproduction user testing. We iteratively collected feedback from users via meetings and surveys.

**Results:**

We conducted 28 meetings with 20 different stakeholder groups. From these stakeholder meetings, we developed 14 low-fidelity prototypes prior to deploying the Discharge Today tool for our pilot study. During the pilot study, stakeholders requested 46 modifications, of which 25 (54%) were successfully executed. We found that most providers who responded to the survey reported that the tool either saved time or did not change the amount of time required to complete their discharge workflow (21/24, 88%). Responses to open-ended questions offered both positive feedback and opportunities for improvement in the domains of efficiency, integration into workflow, avoidance of redundancies, expedited communication, and patient-centeredness.

**Conclusions:**

Survey data suggest that this electronic discharge readiness tool has been successfully adopted by providers and clinical staff. Frequent stakeholder engagement and iterative user-centered design were critical to the successful implementation of this tool.

## Introduction

Communication across care teams in hospitals is often disjointed, which can lead to delays in care and adverse outcomes and can negatively affect team dynamics [1-4]. Planning for care progression and discharge relies on complex communication across multiple care teams, which are often physically separated from each other [1,5,6]. Discharging patients efficiently and safely continues to challenge health care systems worldwide [7-9]. Delays in discharge have been found to be associated with adverse patient outcomes, including mortality, medical complications such as infections, and impaired mobility or activities of daily living, as well as with slowed patient flow from the emergency department and throughout the hospital; these delays are also associated with increased hospital capacity challenges [7,10-14].

Typical approaches for moving discharge to earlier in the day and improving the flow of hospitalized patients rely on one-way communication mechanisms, static documentation in the electronic health record (EHR), and in-person care team huddles or telephone calls, which often take place on the day a patient is expected to be discharged [2,15-22]. Multidisciplinary rounds are a common workflow in many hospitals during which discharging patients are discussed. However, multidisciplinary rounds often vary in execution across clinical units; some approaches are more or less effective than others, with variable start times, different clinical staff in attendance, different processes for discussing the discharge of patients, and variable perception of effectiveness [23-25]. Many of these solutions rely on processes taking place outside of the EHR and interrupt patient care [26,27].

Effective use of health information technology (HIT) may introduce a degree of standardization to multidisciplinary rounds and huddles, improve discharge communication workflows, and alleviate delays in discharge [28]. Although communication between providers using the EHR is not well studied, data indicate that well-executed communication and collaboration between providers is associated with better patient outcomes, and the application of HIT in specific domains is associated with improved health care quality and safety [29,30].

Tools that enable dissemination of information at both the patient level and team level may provide the greatest utility, as providers and other clinical staff would be able to access information for each individual patient as well as for groups of patients being cared for by a specific team or on a specific floor. Given the success found in the application of HIT in specific domains, such as provider order entry or prescribing of medications [29-32], there is potential for the application of real-time electronic provider-to-provider or provider-to-service communication around the activity of discharge planning.

Addressing the need for a seamless solution to coordinating discharge processes, we developed an innovative tool (the Discharge Today tool) within Epic, the EHR in use at the University of Colorado Hospital, to facilitate communication in real time between hospitalists and other clinical staff regarding discharge readiness and barriers to discharge [2]. We hypothesized that systematic application of stakeholder engagement and workflow analyses as a part of a user-centered design process would lead to a well-designed HIT innovation that would be readily adopted and consistently used by providers and other clinical staff.

## Methods

To guide the design of this tool, we applied several frameworks, including the analytic-deliberative model of stakeholder engagement [33] to enhance our stakeholder engagement efforts, the Coiera communication paradigm [34] to incorporate communication theory, and the Chokshi and Mann process model for user-centered digital development [35] to direct the iterative development of the tool.

Applying the analytic-deliberative model of stakeholder engagement [33], we involved our stakeholder partners, including patients, families and caregivers, clinical staff, clinical leadership, and administrative leadership. The analytic-deliberative model links analysis using information collected and deliberation by stakeholders with the intent of reconciling different viewpoints and making recommendations.

To that end, we met with clinical and administrative staff to gain an understanding of their experiences with the discharge process as well as the communication methods and tools currently used to disseminate information on barriers to discharge and readiness for discharge. We conducted workflow analyses with clinical staff directly involved in discharge communication and care of hospitalized patients. Finally, we engaged with patients who experienced discharge from the hospital through one-on-one discussions with patients and their families or caregivers. Stakeholder engagement to inform user-centered design was imperative to ensure that our Discharge Today tool was successfully integrated into existing workflows such that all clinical staff would use this tool with every patient. However, stakeholder engagement was only one aspect of our systematic approach to user-centered design in a clinical setting.

Similar to other types of computer-supported cooperative work technologies that support asynchronous collaboration, such as email, collaborative creation of documents, technologies designed to capture recommendations, repositories for shared information, and particularly workflow applications, the Discharge Today tool is an asynchronous communication tool [36]. To improve the flexibility, agility, efficiency, and accuracy of communication around discharge, we applied the Coiera communication paradigm [34]. This model describes four stages for communication (task identification, connection, communication, and disconnection) in which errors may occur at any point during the sequence, including how the communication system functions or is used or in the information available to those involved. By supporting asynchronous collaboration, building feedback loop capabilities, and implementing user role–dependent functionality, the Discharge Today tool reduces inefficiencies and, potentially, errors in the delivery of health care during the discharge process.

Using the Chokshi and Mann process model for user-centered digital development [35], we applied the four phases described with a continuous feedback loop between Discover, Define, Develop, and Deliver. Phase one requires understanding the concepts and processes associated with the work being done, and phase two involves engaging with users to understand how they would use a tool and observing users in a laboratory environment before going live using two specific methods: “think-aloud” and “near-live” [35]. Phases three and four involve iterative development, testing, and optimization of a tool in the setting where the work is actively being done.

Using the methods described in this model, we were able to identify any fundamental incompatibilities between the EHR and typical clinical workflows, which are potential points of failure for provider-facing innovations. In addition, this model helped guard against overdesign of the tool to accommodate workflows, which can actually inhibit adoption.

As a part of our stakeholder engagement process, we applied a novel strategy, brainwriting premortem [37], to specifically engage stakeholders in identifying potential barriers that we might encounter when implementing the discharge readiness functionality in the EHR. The brainwriting premortem exercise was designed by researchers to rapidly stimulate ideas of ways in which an intervention or tool could fail in a focus group setting. This exercise has been found to be an efficient method for engaging stakeholders and generating feedback, specifically because it is designed to imbue a sense of psychological safety among participants [37]. During this exercise, participants were asked to write down all the reasons each of them could think of that would cause this tool to fail. This process was repeated iteratively, with stakeholders adding ideas to existing pages until no new ideas emerged. Upon completion of the exercise, the pages were collected and the content was collated later for consideration by the project team.

Following multidisciplinary stakeholder meetings and the brainwriting premortem exercise, we constructed the first of 14 low-fidelity prototypes. These prototypes were presented on paper to stakeholders for feedback and revision. The EHR application analysts building this tool provided guidance regarding the capabilities and limitations of the existing EHR functionality.

**Figure 1:**
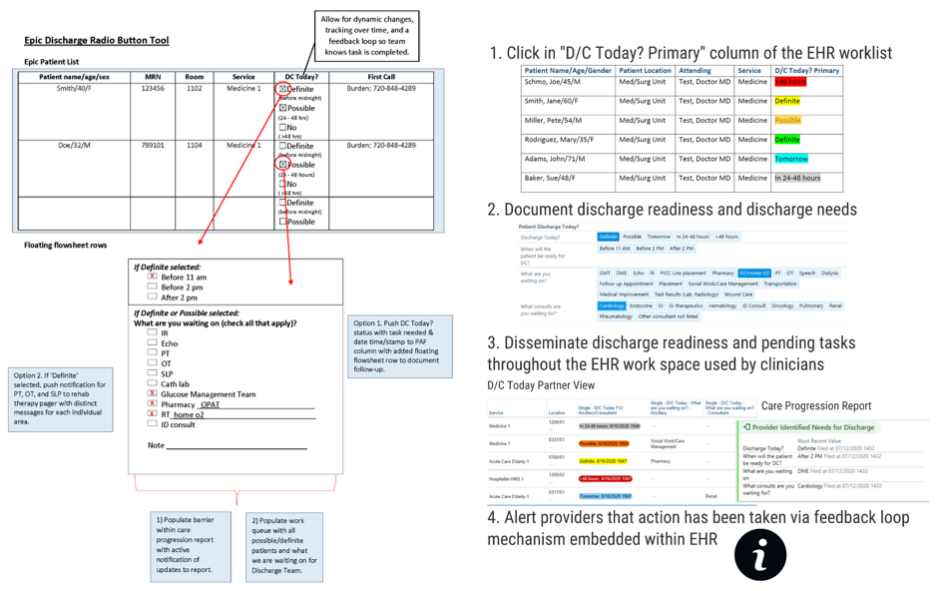
Final low-fidelity prototype prior to EHR development and the Discharge Today tool (demonstration only, no protected health information).

Using the final low-fidelity prototype produced, the Discharge Today tool was constructed in the test EHR environment (Figure 1). We convened “think-aloud” sessions with users from the Division of Hospital Medicine for two purposes. First, we asked users to interact with the tool following minimal instructions and using a modified cognitive task analysis approach [38], while we made note of challenges users encountered or questions asked. This information was used to inform both revisions made to the tool and instructions developed for users. Second, we asked users to talk about their perceptions of the tool, specifically its utility and usability, as they interacted with the tool. This feedback informed modifications made to the tool. Following these sessions, we transitioned to “near-live” testing, in which we conducted preproduction user testing with both hospital medicine providers and ancillary department staff using real patient data and updated instructions. The purpose of this testing was to identify any components of the tool that were not functioning as intended prior to transitioning to the pilot test.

Following any changes or additions to the Discharge Today tool, functionality testing took place in the test EHR environment with a secondary validation stage in a shadow EHR environment with real patient data on a set delay. In addition, the end users each tested any revision or addition to the functionality in the test EHR environment prior to moving updates to production. Monitoring of the functionality of the tool occurred via periodic testing of the tool in both the test EHR environment and the production environment to isolate issues with the tool that were not otherwise identified prior to the go-live phase. In addition, feedback was solicited from end users to identify issues that became apparent during clinical work. We approached clinical staff in their workplaces to obtain real-time feedback on the functionality of the tool.

Surveys were conducted following the final month of the pilot phase using Research Electronic Data Capture (REDCap), a secure, web-based application for building and managing web-based surveys and databases [39]. Physicians, advanced practice providers, nurses, care management staff, and other clinical staff were asked to complete surveys regarding the usability of the Discharge Today tool and their experience with it. The factors chosen for evaluation, including time required to use the tool, accuracy of data collected via the tool, and helpfulness of the tool, were selected based on stakeholder feedback from both providers and other clinical staff.

## Results

During the Discover and Define stage of development, applying the analytic-deliberative model, we engaged with 20 different stakeholders in 28 separate meetings across disciplines and settings, including care managers, nurse managers, patients and caregivers, an established, university-based patient advisory panel, and EHR builders and consultants. We also met several times with clinical directors, advanced practice providers, and physicians from departments of hospital medicine, infectious diseases, cardiology, endocrinology, hematology, pulmonary/critical care, and nephrology. Finally, we met multiple times with clinical staff and managers from respiratory therapy, rehabilitation services (specifically occupational, physical, and speech therapy), interventional radiology, pharmacy, glucose management, echocardiography, the heart and vascular team, and dialysis (Table 1).

**Table 1:** Key stakeholders and their engagement activities.

Key stakeholders	Engagement activities
Patients	1 Patient Advisory Panel meeting 10 telephone conversations
Hospital medicine providers	2 lunch meetings 1 avoidable delay survey 1 user testing session 2 usability and experience surveys
Nursing staff	2 meetings 2 usability and experience surveys
Case management/social work staff	2 meetings 1 usability and experience survey
Physical therapy/occupational therapy/speech-language pathology staff	3 meetings 1 usability and experience survey
Glucose management team members	1 meeting
Pharmacy staff	2 meetings 1 usability and experience survey
Respiratory therapy staff	3 meetings 1 usability and experience survey
Echocardiography staff	2 meetings
Interventional radiology staff	1 meeting
Department of Medicine clinical directors	1 meeting
Infectious disease staff	2 meetings
Cardiology staff	1 meeting
Endocrinology staff	1 meeting
Hematology staff	1 meeting
Pulmonary services staff	1 meeting
Renal medicine staff	1 meeting

**Figure 2:**
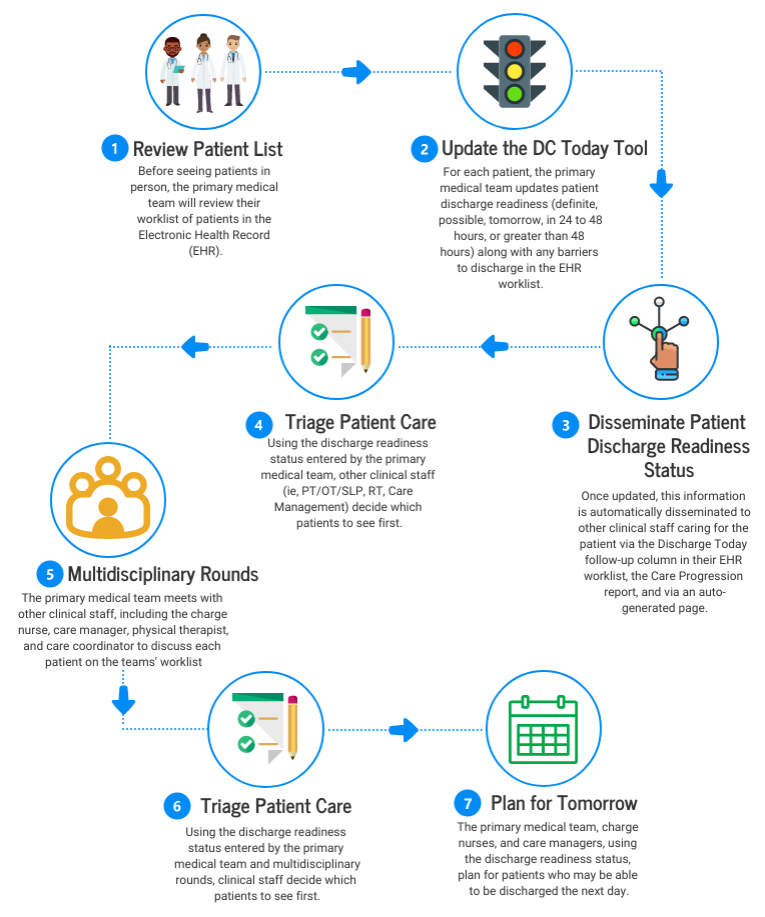
User journey of the patient discharge workflow. DC Today: Discharge Today; OT: occupational therapy; PT: physical therapy; RT: respiratory therapy; SLP: speech-language pathology.

During these meetings, we discussed the stakeholders’ experiences with the discharge process, what went well and what could be improved, and their current workflow related to discharge. We observed clinical staff interacting with the EHR to map how different staff providing care to patients used EHR functionalities and how the Discharge Today tool might best be integrated. Using the information gathered during conversations with and observation of stakeholders, we constructed a user journey to illustrate how the Discharge Today tool might best be integrated with existing workflows and what might be changed (Figure 2).

To work as designed, using guidance provided by the stakeholders involved in our user-centered design process, we developed a framework for our Discharge Today tool, encompassing the following functions and operational processes. First, the tool must populate a list of patients with information from designated data sources and display the results on a user interface dashboard for provider access. Second, the tool must be accessible from the customizable patient worklist available in the provider workflow whenever a provider logs into the EHR. Third, the discharge readiness status for each patient on a provider’s list must be displayed with color-coding (green if the patient is a definite discharge with a discharge order, yellow if the patient is a definite discharge without a discharge order, orange if the patient is a possible discharge this day, blue if the patient could go home tomorrow, red if the patient is not going home this day, and gray if the patient is expected to go home in the next 24 to 48 hours). Fourth, data collected from primary team providers each morning via the Discharge Today tool must be pushed automatically through three different processes that are integrated seamlessly with existing clinical workflows: the EHR patient worklists via the Discharge Today follow-up column, the Care Progression report, and an auto-generated page. Finally, through a feedback mechanism implemented such that when staff from ancillary departments such as respiratory therapy (RT), physical therapy (PT), occupational therapy (OT), and speech-language pathology (SLP) document patient care in the EHR using their standard workflow, the primary team provider who originally reported a requirement from these ancillary departments must be alerted that something has changed, creating a feedback loop within the EHR. To alert providers using the Discharge Today tool, an icon indicating new information is populated in the Discharge Today tool column displayed in the provider’s list. Combining this functionality creates a tool that enables real-time communication among care team members via the EHR.

All data collected by the Discharge Today tool are stored in the transactional database of the EHR at the level of the patient hospital encounter. This supports real-time use, functional processes, and dashboard population. The tool populates a list of patients managed by individual providers with patient attributes, encounter attributes, provider attributes, and discharge readiness status, timing, and barriers into a user interface dashboard. Providers interact with their patient list in the dashboard and make item entries for each patient from structured category lists (Table 2). The data entered into the tool by the primary team provider populate the “Provider Identified Needs for Discharge” section of the Care Progression report used by providers, nursing staff, and care management staff to view the overall care of the patient during the hospitalization. The data entered also autopopulate a Discharge Today Follow-up column that is used as part of the provider’s patient worklists by consulting teams (eg, cardiology, endocrine, and gastrointestinal) and ancillary services (eg, RT, pharmacy, OT, PT, SLP, and wound care). Finally, for OT, PT, and SLP, an autogenerated page is sent that is populated with patient and discharge barrier data when a patient is identified as a definite discharge waiting on a final evaluation from these services.

**Table 2:** Discharge Today data elements and sources in the electronic health record.

Data element	Data source/location
Patient attributes	Patient record
Encounter attributes	Hospital encounter record
Provider attributes	Provider record
Discharge probability categories	Transactional database tables
User interface highlight colors	Code extension
Discharge timing categories	Transactional database tables
Discharge barriers	Transactional database tables/alert criteria
Discharge follow-up comments	Transactional database tables

During the Develop and Deliver phase, from March 5 to July 31, 2019, we conducted iterative development, testing, and optimization of the Discharge Today tool while in use by Hospital Medicine advanced practice providers and physicians. During this phase, stakeholders requested 46 modifications, with 85% of these requests occurring in the first two months of the pilot study. Of the 46 modifications, 11 (24%) were set aside due to existing limitations in EHR functionality, and 10 (22%) were considered to have insufficient utility or potential for overdesign and were thus not pursued. A total of 25/46 modifications (54%) were successfully executed, and 3 of the 25 modifications (12%) were fully implemented after the end of the pilot period (Table 3).

**Table 3:** Modifications to the Discharge Today tool (N=25).

Date requested (2019)	Request	Date fully modified (2019)
March 7	Rename columns to help with clarity when providers are wrenching them in	March 8
March 11	If a provider reselects “possible,” “definite,” or “no,” reset the branching logic	March 14
March 17	PT^a^/OT^b^/SLP^c^ pages are sent out when selected, with lockout if more than one page is selected within 12 hours	March 8
April 1	Update names of columns to be less confusing for wrenching in or display in larger patient lists	April 8
March 5	Add Transportation as a barrier	April 12
March 6	Add PICC^d^ Line Placement as a barrier	April 12
March 8	Add a way to indicate future discharge (ie, in 24-48 hours)	April 12
March 12	Add DME^e^ as a barrier	April 12
March 12	Update RT^f^ barrier to Home O_2_	April 12
March 12	Update the Social Work barrier to Social Work/Care Management	April 12
March 12	Add “Other consultant not listed” as a barrier	April 12
March 12	Update pager system to allow a page once every 12 hours	April 12
April 19	Combine PT and OT pager numbers	April 26
April 24	Indicate in the page set to PT/OT which discharge selection was made (“Possible” or “Definite”)	April 26
March 11	Reset column after 3 days	May 23
March 11	Automatically update to definite (green) when a discharge order is placed	May 23
April 11	Change the order of the barrier selections	May 23
April 12	New column to display barrier selections from the Discharge Today Primary column	May 23
April 12	Make the “In 24-48 hours” selection gray in color	May 27
March 5	Develop a feedback loop	June 24
April 26	Add Test Results (Laboratory, Radiology) as a barrier	June 27
June 14	Add Wound Care as a barrier	June 27
May 7	Add fields to capture more information about PT/OT barriers	July 30
July 2	Change “No” to “>48 hours”	September 27
March 15	Add option to select for anticipated discharge tomorrow	December 3

^a^PT: physical therapy.

^b^OT: occupational therapy.

^c^SLP: speech-language pathology.

^d^PICC: peripherally inserted central catheter.

^e^DME: durable medical equipment.

^f^RT: respiratory therapy.

We found that most providers who responded to the usability and experience survey (21/24, 88%) reported that the tool either shortened or did not change the amount of time required to complete the discharge workflow. Of the nursing, care management, and other clinical staff surveyed who reported using the Discharge Today tool during the pilot study (34/67, 51%), all felt that the tool either shortened or did not change the amount of time required to complete their workflows. In addition, a majority of ancillary staff who completed the survey reported that they believed that hospitalists were updating the discharge information (26/34, 77%), that the information was accurate (22/34, 65%), and that the information was helpful (32/34, 94%). These data suggest that the Discharge Today tool was successfully adopted by providers and other clinical staff (Table 4).

**Table 4:** Provider (n=24) and clinical staff (n=67) responses to the survey on usability and experience of the Discharge Today tool following the pilot implementation period.

Question				Response, n (%)
**Providers (n=24)**				
	**Please select the ways in which you used the discharge tool (check all that apply).**			
		Entered/updated discharge information in patient list column	21 (88)	
		Viewed discharge information in patient list column	13 (54)	
		Viewed discharge information in the care progression report	3 (13)	
		Determine order of rounds, prioritizing early discharges	1 (4)	
	**For what percentage of your patients did you use the tool?**			
		0%-25%	0 (0)	
		26%-50%	5 (21)	
		51%-75%	3 (13)	
		76%-100%	16 (67)	
	**When did you utilize the tool the most?**			
		Beginning of shift	21 (88)	
		Middle of shift	5 (21)	
		End of shift	6 (25)	
	**How did the tool affect your discharge workflow?**			
		Saved time	6 (25)	
		Added time	3 (13)	
		Did not change	15 (63)	
**Clinical stall** **(n=67)**				
	**Did you use the Discharge Today – Follow-up Ancillary/Consultant tool over the last month?**			
		Yes	34 (51)	
		No	33 (49)	
	**Please select the ways in which you used the discharge tool.**			
		Viewed discharge information in my clinical workflow	31 (91)	
		Contacted hospitalist who entered information in Epic	5 (15)	
		Viewed discharge information in the care progression report	14 (41)	
	Do you feel hospitalists are completing and updating the discharge information?		26 (77)	
	Did you find the information accurate?		22 (65)	
	Did you find the information helpful?		32 (94)	
	**How did the tool affect your discharge workflow?**			
		Saved time	21 (62)	
		Added time	0 (0)	
		Did not change	13 (38)	
	Do you find the tool helpful?		31 (91)	
	**What prevented you from using the tool?**			
		Discharge information not completed by hospitalists	6 (18)	
		Information not updated/accurate	7 (21)	
		Lack of time	5 (15)	
		Lack of knowledge	20 (61)	
		Forgot/overlooked	3 (9)	
		Chose not to	1 (3)	
		Other	4 (12)	

We also collected qualitative usability and experience data from hospital medicine providers and clinical staff following the pilot implementation period using open-ended questions in the REDCap survey. Themes were derived from responses provided to five open-ended questions included in the survey. Free text responses were coded, and a synthesis of the results emerging from the responses to each of the open-ended questions was summarized (Table 5).

Responses were categorized into five themes, namely efficiency, integration into workflow, redundancies avoided, expedited communication, and patient-centered outcomes. The data provided both positive feedback and opportunities for improvement.

**Table 5:** Qualitative usability and experience data from hospitalists and other clinical staff following pilot implementation of the Discharge Today tool.

Theme	Quotes	
	Positive feedback	Opportunities for improvement
Efficiency	“Noticed quick responses from PT/OT for evaluation which expedited discharge.” “I think it is quick and hopefully as all ancillary staff learn to utilize it can continue to improve discharge times.”	“Not all teams are utilizing the tool yet.”
Integration into workflow	“Well integrated into my existing workflow.”	“Sometimes the options available to explain what is holding up a discharge does not apply…would be nice to have an “other” comment box.”
Avoidance of redundancy	“In theory, it should avoid redundancies and emphasize the hold up to discharges…If nurses know we are consistently updating this it would help eliminate unnecessary pages.”	“Other services/staff learning to utilize it in their workflows.” “Some ancillary services are still utilizing old workflows.”
Expedited communication	“It is nice to be able to state what would be potentially holding up the discharge and not have to call those services/departments directly.”	“A little more feedback about what is happening as we click these things (like a little small font blurb).”
Patient-centered outcomes	“Per the DC tool knew [the patient was] going to be going home in the next day or two. I was able to decide on a DC plan and send the prescriptions to the pharmacy for fill. Low [*sic*] and behold, the insulin prescribed was not covered so we were able to revise the plan well before day of DC therefore avoiding a delay.”	“Would it be possible that a checklist could be given to the patient? Allowing patient to follow the process…an opportunity to ask questions?”

## Discussion

The important findings of this work are (1) providers, hospital clinical staff, and patients are willing to serve as stakeholders to help guide the user-centered design of an EHR-based tool and (2) stakeholder engagement during preimplementation, throughout implementation, and into postimplementation results in positive feedback and substantial adoption by clinical staff.

We applied communication theory to the design of this tool with the intent of fostering interdisciplinary discharge communication and teamwork. Communication across care teams and improved interdisciplinary care has been recognized as an important factor for high-quality patient-centered care and for high-functioning teams. Studies have shown that when care teams communicate better, efficiency outcomes are improved [18]. Patients have also expressed a need for the clinical staff caring for them to communicate with each other more effectively [40].

Studies exploring the use of the EHR for discharge planning have been limited to static electronic reports constructed from EHR data elements, including barriers to discharge documented at admission, care management data, and discharge criteria [19], or other targeted interventions, such as improving discharge summaries for patients or medication reconciliation at discharge [20,21,32,41]. In contrast, our Discharge Today tool was designed to capture and disseminate patient discharge readiness in a real-time, dynamic way, as opposed to merely reporting static discharge information via standard report functionality.

Tyler et al [19] reported developing and implementing an EHR-based discharge readiness report for medical and medical subspecialty patients that provides a summary of information related to patient discharge. As with our tool, this report was easily accessible and readily adopted by clinical staff. Researchers from the University of Wisconsin Hospital and Clinics described designing an EHR-based discharge summary template that was successfully adopted by clinicians hospital-wide [21]. Similar to these other projects designed to improve discharge communication and workflow, our Discharge Today tool was readily adopted by both providers and other clinical staff.

Although common quality improvement tactics, such as identifying champions, Plan-Do-Study-Act cycles, and process mapping, are valuable tools, developing and implementing HIT innovations necessitates frameworks and methods that are specifically designed for HIT. To engage hospitalists, nurses, other clinical staff, patients, families and caregivers, and hospital leadership, we met with 20 different stakeholder groups to obtain feedback about the design and functionality of the tool. Following this engagement process, we made improvements, implemented a pilot tool, and assessed discharge processes and both provider and clinical staff experience with the tool. To guide the development and implementation of our pilot Discharge Today tool, we chose to apply the analytic-deliberative model of stakeholder engagement [33] and the Chokshi and Mann process model for user-centered digital development [35].

Our approach to stakeholder engagement and user-centered design had a number of strengths. We deliberately, proactively applied established frameworks to guide both our stakeholder engagement process and the process of designing our tool. In addition, we leveraged existing functionality in our EHR to create an innovative discharge communication tool based on a design framework developed in collaboration with our stakeholders. Finally, this discharge communication tool facilitates real-time communication across hospital clinical staff, reducing reliance on static communication tools or interruptions to clinical care.

Our approach had a few limitations. We were unable to identify stakeholders in every clinical area of the hospital with whom communication about patient discharge readiness or barriers may occur. In addition, limitations to functionality of the EHR at the time of the development of this tool restricted the development of feedback loops to discharge barriers related to physical therapy, occupational therapy, speech therapy, and respiratory therapy rather than across all clinical areas. We continue to work with hospital leadership to fully integrate the Discharge Today tool with other initiatives implemented to improve discharge processes, improve patient flow, and alleviate capacity problems. Finally, as this tool expands in scale, future work will begin to assess how this type of tool (and future modifications thereof) affects quality measures such as patient experience, teamwork, and potentially readmissions.

By using a deliberate and collaborative stakeholder engagement process, we obtained commitments from numerous key stakeholders to participate in the design and testing of our EHR discharge readiness tool. The tool has been implemented for clinical use, and we have conducted an extensive evaluation of the implementation and effectiveness of the tool from a multistakeholder perspective. Survey data collected from Hospital Medicine providers and ancillary clinical staff suggest that the tool has been successfully adopted by clinical staff.

## Abbreviations

EHR: electronic health record
HIT: health information technology
OT: occupational therapy
PT: physical therapy
REDCap: Research Electronic Data Capture
RT: respiratory therapy
SLP: speech-language pathology
